# A novel and non-invasive method for DNA extraction from dry bee specimens

**DOI:** 10.1038/s41598-022-15595-8

**Published:** 2022-07-08

**Authors:** Giovanni Cilia, Simone Flaminio, Marino Quaranta

**Affiliations:** CREA Research Centre for Agriculture and Environment, Via di Corticella 133, 40128 Bologna, Italy

**Keywords:** Molecular biology, Zoology

## Abstract

In recent years molecular techniques have been used on museum material as integrative support for classic taxonomy. This cumulative systematics approach is especially for rare or extinct specimens, and genetic analysis may be useful to discern information that is not possible to glean from live materials or morphology. To date, the extraction of DNA required at least a partial destruction of the specimens, which is not possible for all individuals, especially the types. In this study, we described a novel method to extract mitochondrial DNA (mtDNA) from pinned museum bee individuals to avoid any external morphological damage. This method was able to amplify the mtDNA *Cytochrome C oxidase subunit I* (*COI*) gene in bee samples collected up to 27 years ago. We tested the efficacy of this method on 72 preserved be specimens belonging to nine species among four families, it could be used on many museums’ rare and/or extinct bee species because it does not provide external morphological damages. The method could be helpful for providing ecological, taxonomic, and phylogenetic information about specimens preserved in museum collections.

## Introduction

Insects are currently facing global decline^1^, to better understand their historic patterns and assess biodiversity without further depleting populations, there is a necessity to turn to museums. Genetic and genomic techniques and their applications have proved to be a useful complement to morphological taxonomy^2^. The array of molecular approaches is effective in delineating evolutionary boundaries, it does not replace the critical role of classic morphological taxonomy^3^. Indeed, classic taxonomy remains a fundamental discipline at the basis of biological sciences, which the advent of molecular approaches has expanded upon and enriched. For example, the molecular taxonomy has facilitated the description of delimitating species boundaries and for phylogenetic reconstruction^4^, understanding the species boundaries^5,6^ and reconstructing the species lineage^7–9^.

Insects represent 40% of all living species, this massive biodiversity has made museums a critical place for studying them, as they foster historic material on a global scale^10^. Molecular taxonomy, as mentioned above, has been identified as a valid instrument for museum material, making the scope of knowledge more accessible^11^. Recently, the molecular studies to support classical taxonomy have constantly increased but they are focused especially on individuals collected for genetic analysis and preserved in a specific way to not degrade the DNA (i.e. avoid the use of ethyl acetate)^7^. Unfortunately, this does not allow the use of rare or extinct species and type material. The current approaches to DNA extraction require the total or, at least, partial destruction of the individuals^3,7,12^, approaches that should not be used for unique specimens or specimens attributable to voucher collections. To defeat these limitations, in 2007 Gilbert and colleagues developed a method to extract DNA on museum specimens without external morphological damages^13^. This method is very successful for beetles because they are covered by a robust, hairless exoskeleton, but is unsuitable for bees, due to the treatment with digestion buffer which leads to depigmentation of hair, an important morphological character for bee taxonomy. Our project aimed to test the above-mentioned Gilbert et al.^13^ method of non-invasive DNA extraction on bee specimens, a group that has 20,000 species and is critical for biodiversity and ecosystem functions.

## Results

**Figure 1:**
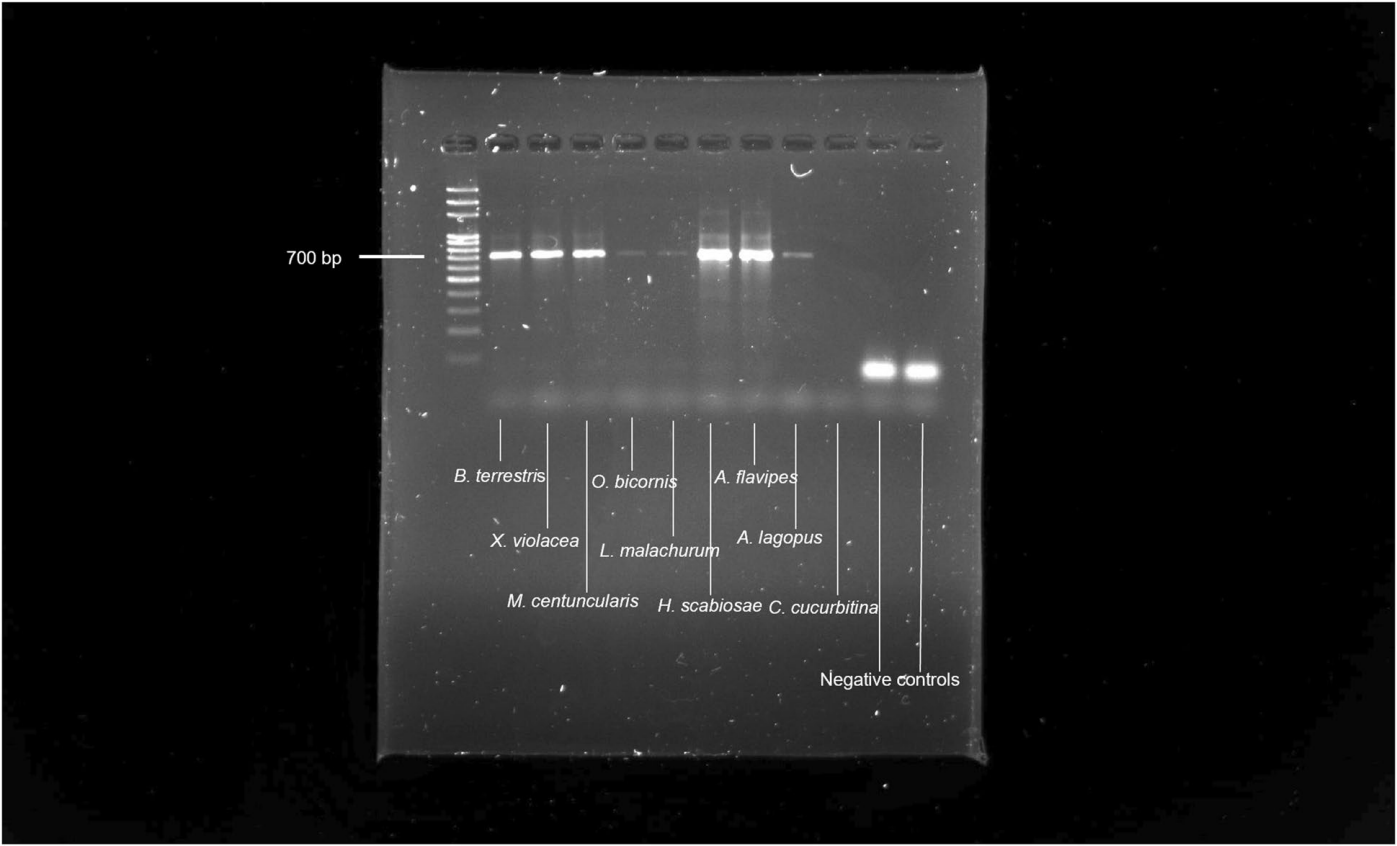
Gel electrophoresis of PCR performed on DNA amplified from the investigated bee species. As a negative control double distilled RNase-DNase-free water. Light bands also could mean low DNA concentration.

mtDNA was successfully amplified (Fig. 1) and sequenced from 8 of the 9 bee species investigated, regardless of when they were collected (Table 1). The only species we could not amplify DNA from was *Ceratina cucurbitina* (Rossi, 1792).

**Table 1:** Details of bee individuals analyzed in this investigation.

ID	Species	Geographic origin	Year of sampling	Amplification mtDNA	Sequence length	Accession number
1	*Bombus terrestris* (Linneaus., 1758)	Italy, Emilia Romagna	2021	Yes	268 bp	OL986022
2	*Bombus terrestris* (Linneaus., 1758)	Italy, Emilia Romagna	2021	Yes	244 bp	OL986023
3	*Bombus terrestris* (Linneaus., 1758)	Italy, Emilia Romagna	2018	Yes	241 bp	OL986024
4	*Bombus terrestris* (Linneaus., 1758)	Italy, Emilia Romagna	2017	Yes	220 bp	OL986025
5	*Bombus terrestris* (Linneaus., 1758)	Italy, Emilia Romagna	2011	Yes	254 bp	OL986026
6	*Bombus terrestris* (Linneaus., 1758)	Italy, Emilia Romagna	2011	Yes	220 bp	OL986027
7	*Bombus terrestris* (Linneaus., 1758)	Italy, Umbria	1994	Yes	245 bp	OL986028
8	*Bombus terrestris* (Linneaus., 1758)	Italy, Sicily	2002	Yes	213 bp	OL986029
9	*Xylocopa violacea* (L., 1758)	Italy, Campania	2021	Yes	413 bp	OL966999
10	*Xylocopa violacea* (L., 1758)	Italy, Emilia Romagna	2021	Yes	317 bp	OL967010
11	*Xylocopa violacea (L., 1758)*	Italy, Sardinia	2017	Yes	367 bp	OL979169
12	*Xylocopa violacea* (L., 1758)	Italy, Emilia Romagna	2018	Yes	354 bp	OL979170
13	*Xylocopa violacea (L., 1758)*	Italy, Emilia Romagna	2011	Yes	329 bp	OL979171
14	*Xylocopa violacea* (L., 1758)	Italy, Emilia Romagna	2011	Yes	340 bp	OL979172
15	*Xylocopa violacea* (L., 1758)	Italy, Ligura	2003	Yes	355 bp	OL979173
16	*Xylocopa violacea* (L., 1758)	Greece, Thessaly	1992	Yes	335 bp	OL979174
17	*Ceratina cucurbitina* (Rossi, 1792)	Italy, Campania	2021	No	nd	na
18	*Ceratina cucurbitina* (Rossi, 1792)	Italy, Campania	2021	No	nd	na
19	*Ceratina cucurbitina* (Rossi, 1792)	Italy, Emilia Romagna	2017	No	nd	na
20	*Ceratina cucurbitina* (Rossi, 1792)	Italy, Emilia Romagna	2016	No	nd	na
21	*Ceratina cucurbitina* (Rossi, 1792)	Italy, Emilia Romagna	2011	No	nd	na
22	*Ceratina cucurbitina* (Rossi, 1792)	Italy, Emilia Romagna	2011	No	nd	na
23	*Ceratina cucurbitina* (Rossi, 1792)	Italy, Latium	2006	No	nd	na
24	*Ceratina cucurbitina* (Rossi, 1792)	Italy, Latium	2002	No	nd	na
25	*Osmia bicornis* (Linneaus, 1758)	Italy, Veneto	2021	Yes	480 bp	OL979211
26	*Osmia bicornis* (Linneaus, 1758)	Italy, Veneto	2021	Yes	367 bp	OL979212
27	*Osmia bicornis* (Linneaus, 1758)	Italy, Tuscany	2017	Yes	322 bp	OL979213
28	*Osmia bicornis* (Linneaus, 1758)	Italy, Tuscany	2017	Yes	370 bp	OL979214
29	*Osmia bicornis* (Linneaus, 1758)	Italy, Emilia Romagna	2011	Yes	211 bp	OL979215
30	*Osmia bicornis* (Linneaus, 1758)	Italy, Emilia Romagna	2011	Yes	288 bp	OL979216
31	*Osmia bicornis* (Linneaus, 1758)	Italy, Umbria	2003	Yes	308 bp	OL979217
32	*Osmia bicornis* (Linneaus, 1758)	Italy, Umbria	2000	Yes	335 bp	OL979218
33	*Megachile centuncularis* (Linneaus, 1758)	Italy, Emilia Romagna	2020	Yes	261 bp	OL981351
34	*Megachile centuncularis* (Linneaus, 1758)	Italy, Emilia Romagna	2020	Yes	238 bp	OL981352
35	*Megachile centuncularis* (Linneaus, 1758)	Italy, Emilia Romagna	2018	Yes	291 bp	OL981353
36	*Megachile centuncularis* (Linneaus, 1758)	Italy, Emilia Romagna	2018	Yes	314 bp	OL981354
37	*Megachile centuncularis* (Linneaus, 1758)	Italy, Emilia Romagna	2011	Yes	303 bp	OL981355
38	*Megachile centuncularis* (Linneaus, 1758)	Italy, Emilia Romagna	2011	Yes	236 bp	OL981356
39	*Megachile centuncularis* (Linneaus, 1758)	Italy, Latium	1997	Yes	300 bp	OL981357
40	*Megachile centuncularis* (Linneaus, 1758)	Italy, Umbria	1996	Yes	311 bp	OL981358
41	*Andrena flavipes* (Panzer, 1799)	Italy, Emilia Romagna	2021	Yes	215 bp	OL982531
42	*Andrena flavipes* (Panzer, 1799)	Italy, Campania	2021	Yes	180 bp	OL982532
43	*Andrena flavipes* (Panzer, 1799)	Italy, Emilia Romagna	2018	Yes	205 bp	OL982533
44	*Andrena flavipes* (Panzer, 1799)	Italy, Emilia Romagna	2018	Yes	209 bp	OL982534
45	*Andrena flavipes* (Panzer, 1799)	Italy, Emilia Romagna	2011	Yes	186 bp	OL982535
46	*Andrena flavipes* (Panzer, 1799)	Italy, Emilia Romanga	2011	Yes	196 bp	OL982536
47	*Andrena flavipes* (Panzer, 1799)	Italy, Umbria	1996	Yes	203 bp	OL982537
48	*Andrena flavipes* (Panzer, 1799)	Italy, Tuscany	1994	Yes	172 bp	OL982538
49	*Andrena lagopus* (Fabricius, 1775)	Italy, Emilia Romagna	2020	Yes	291 bp	OL981466
50	*Andrena lagopus* (Fabricius, 1775)	Italy, Emilia Romagna	2020	Yes	286 bp	OL981467
51	*Andrena lagopus* (Fabricius, 1775)	Italy, Emilia Romagna	2018	Yes	207 bp	OL981468
52	*Andrena lagopus* (Fabricius, 1775)	Italy, Emilia Romagna	2018	Yes	205 bp	OL981469
53	*Andrena lagopus* (Fabricius, 1775)	Italy, Emilia Romagna	2011	Yes	224 bp	OL981470
54	*Andrena lagopus* (Fabricius, 1775)	Italy, Emilia Romagna	2011	Yes	215 bp	OL981471
55	*Andrena lagopus* (Fabricius, 1775)	Italy, Latium	1996	Yes	214 bp	OL981472
56	*Andrena lagopus* (Fabricius, 1775)	Italy, Latium	1996	Yes	230 bp	OL981473
57	*Lasioglossum malachurum* (Kirby, 1802)	Italy, Piedmont	2021	Yes	236 bp	OL984023
58	*Lasioglossum malachurum* (Kirby, 1802)	Italy, Piedmont	2021	Yes	226 bp	OL984024
59	*Lasioglossum malachurum* (Kirby, 1802)	Italy, Emilia Romagna	2018	Yes	197 bp	OL984025
60	*Lasioglossum malachurum* (Kirby, 1802)	Italy, Emilia Romagna	2018	Yes	236 bp	OL984026
61	*Lasioglossum malachurum* (Kirby, 1802)	Italy, Tuscany	2011	Yes	204 bp	OL984027
62	*Lasioglossum malachurum* (Kirby, 1802)	Italy, Tuscany	2011	Yes	218 bp	OL984028
63	*Lasioglossum malachurum* (Kirby, 1802)	Italy, Sicily	2000	Yes	201 bp	OL984029
64	*Lasioglossum malachurum* (Kirby, 1802)	Italy, Latium	1996	Yes	208 bp	OL984030
65	*Halictus scabiosae* (Rossi, 1790)	Italy, Emilia Romagna	2021	Yes	204 bp	OL984254
66	*Halictus scabiosae* (Rossi, 1790)	Italy, Emilia Romagna	2021	Yes	195 bp	OL984255
67	*Halictus scabiosae* (Rossi, 1790)	Italy, Emilia Romagna	2018	Yes	214 bp	OL984256
68	*Halictus scabiosae* (Rossi, 1790)	Italy, Emilia Romagna	2017	Yes	214 bp	OL984257
69	*Halictus scabiosae* (Rossi, 1790)	Italy, Emilia Romagna	2011	Yes	193 bp	OL984258
70	*Halictus scabiosae* (Rossi, 1790)	Italy, Emilia Romagna	2011	Yes	207 bp	OL984259
71	*Halictus scabiosae* (Rossi, 1790)	Italy, Liguria	2004	Yes	207 bp	OL984260
72	*Halictus scabiosae* (Rossi, 1790)	Italy, Liguria	2004	Yes	217 bp	OL9842561

*nd* not detected, *na* not available.

No amplification was obtained from the negative control and no DNA sequence highlighted evidence of contamination or alteration, and their identity was confirmed through the BLAST analysis and the alignment with sequences deposited in the Barcoding of Life Data system (BOLD system) (https://www.boldsystems.org/index.php/).

Sequences were deposited in GenBank (OL961135, OL966966, OL966999, OL967010, OL979169-OL979174, OL979211-OL979218, OL982531-OL982538, OL984023-OL984030; OL986022-OL986029).

**Figure 2:**
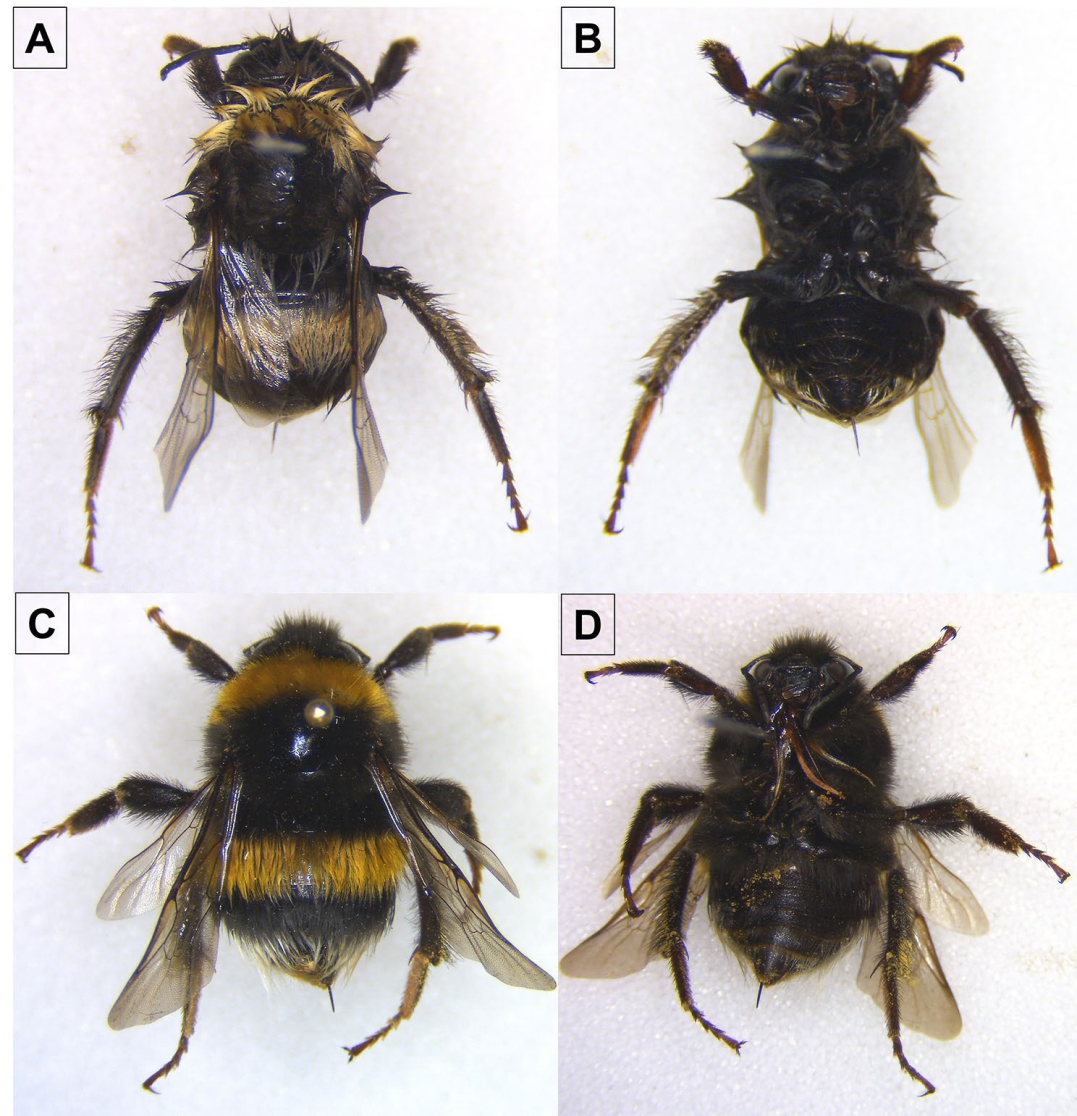
Dorsal and ventral comparison between the post-treatment in two *Bombus terrestris* individuals using the Gilbert et al.^13^ (**A**,**B**) and the here proposed (**C**,**D**) methods.

**Figure 3:**
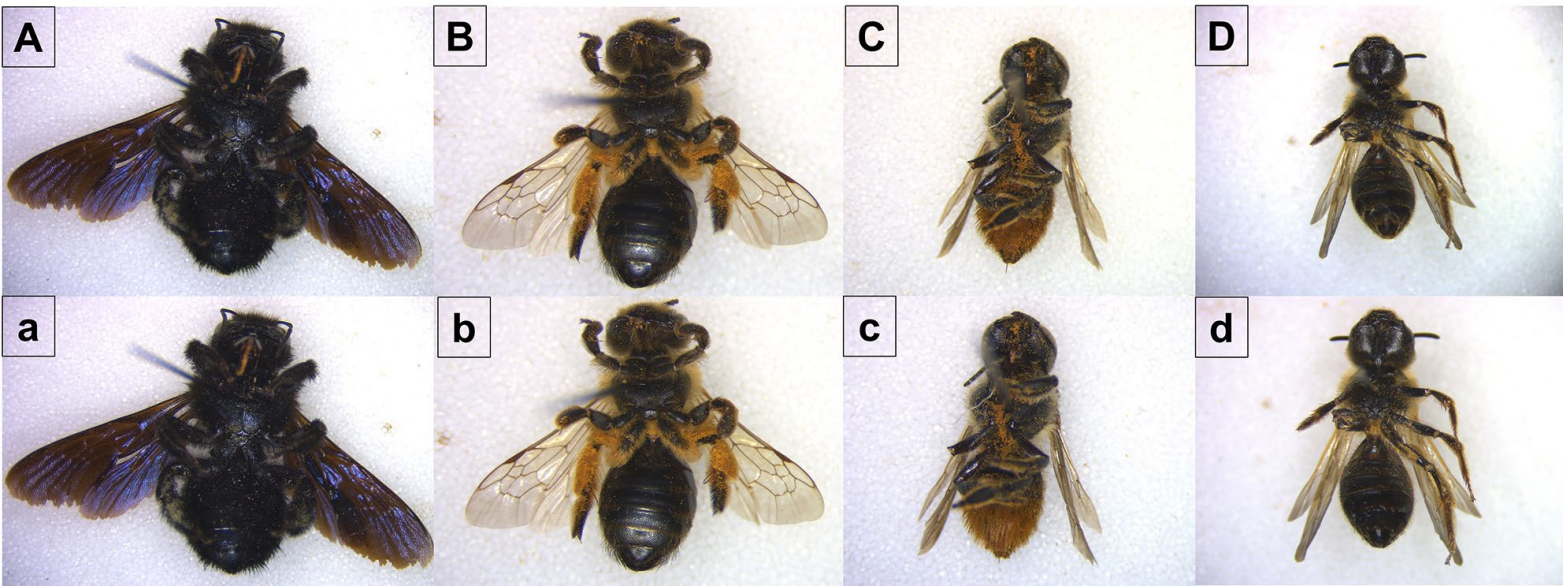
Ventral pictures of four bee individuals before (**A**–**D**) and after (a,b,c,d) the treatment using the swabs soaked with the digestion buffer. (**A**)/a *Xylocopa violacea* (L., 1758) (collected in Emilia-Romagna region in 2011), (**B**)/b *Andrena flavipes* (Panzer, 1799) (collected in Tuscany region in 1994), (**C**)/c *Megachile centuncularis* (L., 1758) (collected in Umbria region in 1996), (**D**)/d *Andrena lagopus* (Fabricius, 1775) (collected in Latium region in 1996).

Current non-destructive methods of total immersion of the individual in buffer (Fig. 2) alters the colouration of the bee's hair and diagnostic characters. Whereas our extraction method using swabs does not exhibit significant external damage or colour change, thus validating its use on important specimens’ the remainder is not necessary as is implied (Fig. 3). To confirm that, all investigated specimens retained all diagnostic characters for recognition at the species level, after careful post-analysis microscopic examination. This supports the potential of the proposed method.

## Discussion

Before this investigation, only a few studies showed evidence for the possibility of extracting DNA from bee specimens non-destructively. These methods required puncturing the exoskeleton, grinding body parts, or immersion in a buffer, all of which result in the destruction of the specimen in some capacity^14^, while the full immersion of the specimens in the digestion buffer^13^. This method did not confer any external damages to the specimens, but is not applicable for hairy insects, like bees, because the digestion buffer causes the depigmentation of hairs. We proposed here a non-invasive method that does not cause any external damages and can be done without removing the entomological pin, decreasing further damages. The method consisting of the full immersion of bee individuals in the buffer was just used to perform the DNA barcoding of a new Megachilidae species, *Trachusa vietnamensis*^15^*.* Although the extraction was successful, it was very complicated to restore the individual to an acceptable initial condition. Several drying processes were necessary to avoid changing the appearance and diagnostic characteristics, which took a total of 3 days. This makes the proposed method an important aid for the molecular identification of bee specimens.

As previously reported, in beetles the digestion buffer acts to liberate the DNA from the mouth, anus, spiracles, sclerites, ectodermal glands, and possibly broken setae, in pinned beetles, the man-made opening in the left elytron and pterothorax^13^. It remains unclear why it has not been possible to extract DNA from *C. cucurbitina* individuals, though it could be related to the size of bee individuals, as it is the smallest investigated species. The fragility along with size could also pose a problem, as in the attempt to not break the specimens, the microbiological swab was passed too gently which did not produce the required results. It is necessary to investigate this question on as many bee species as possible to know the feasibility of the proposed method across taxa.

Although DNA degradation increases over time^16,17^, the mitochondrial DNA remain amplifiable through PCR for a long period^13,17^. For this reason, we were able to extract DNA from investigated individuals, promoting non-destructive methods for bees. On the other hand, the possible DNA degradation can be caused by chemical reactions with ethyl acetate or ethyl alcohol (compounds usually used to kill bees upon collection)^18–20^, which seems to affect DNA integrity and its. This degradation might be probably also linked to no DNA sequences obtained from all investigated *Ceratina* individuals.

The efficacy of this proposed method is also improved by the results obtained from the sequence analysis. Each obtained sequence matched (from 95 to 100% Identity) with *COI* sequences deposited. None of these sequences amplified a *Wolbachia* sp., a problem recently highlighted for the barcoding of bees^21^, which further highlights the robustness of our method.

Given the demand for the application of molecular taxonomy on museum specimens, our method can be effective. The museum collections preserved rare species and individuals collected in habitats that have changed through time, which could give not only important taxonomic and phylogenetic information but also ecological and evolutionary data. The next approach will be to test the method with museum specimens sampled more than 30 years ago, to verify its feasibility for rare old materials. This method minimizes the risk of damaging the specimen, critical for the future of the field.

The proposed methods could increase the taxonomic information on bee individuals preserved in museums and historical entomological collections, as many of these materials are very rare and not yet investigated. Although we considered hairs as a possible source from which DNA was extracted, it cannot be ruled out that this method is also effective for even other arthropods. We have focused mainly on bees since that is our subject of study, but we hope that this method will be useful for the whole entomological research, implementing knowledge on species that are present in rare or even extinct. Due to the high heterogeneity of the insect class, it is impossible to define a single method for DNA extraction from preserved individuals in collections. This, therefore, makes it necessary to test its efficacy across taxa.

## Methods

From the entomological collection of the Research Centre for Agriculture and Environment (CREA-AA), seventy-two dry bee specimens belonging to nine common and widely distributed species among 4 families, between 2021 to 1992, were selected for DNA analysis and sequencing (Table 1). All precautions have been taken to avoid environmental contamination, sterilizing the working tools and the worktop after each processed sample, and molecular works were performed in sterile conditions under a laminar flow hood. A specific digestion buffer was used for the analysis, modified from Gilbert et al*.*^13^, consisting of 5 mM CaCl_2_, 2% sodium dodecyl sulphate (SDS), 65 mM dithiothreitol (DTT), 450 µg/ml proteinase K, 150 mM Tris buffer pH 8 and 100 mM NaCl.

**Figure 4:**
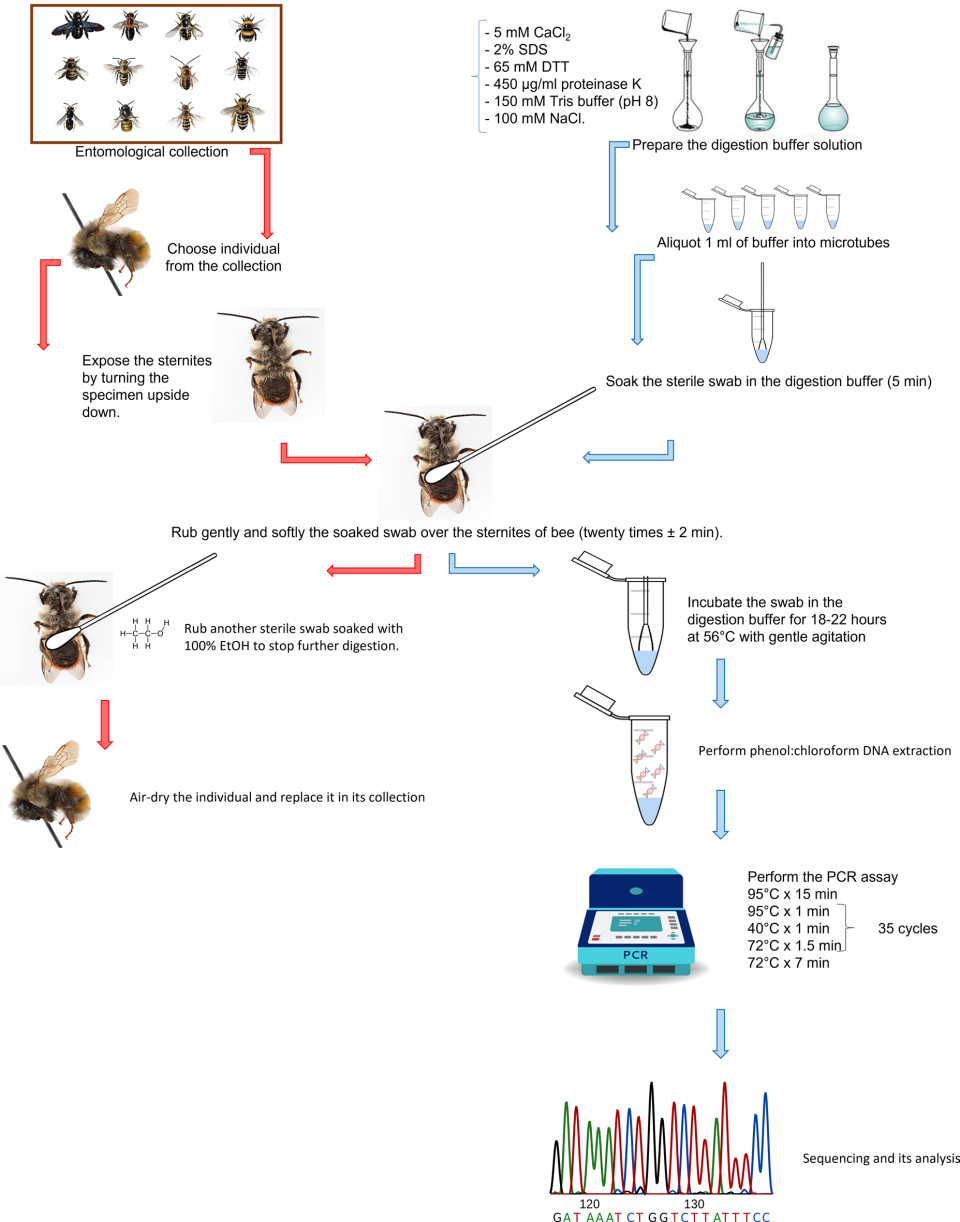
Schematic description of the experimental procedures.

**Figure 5:**
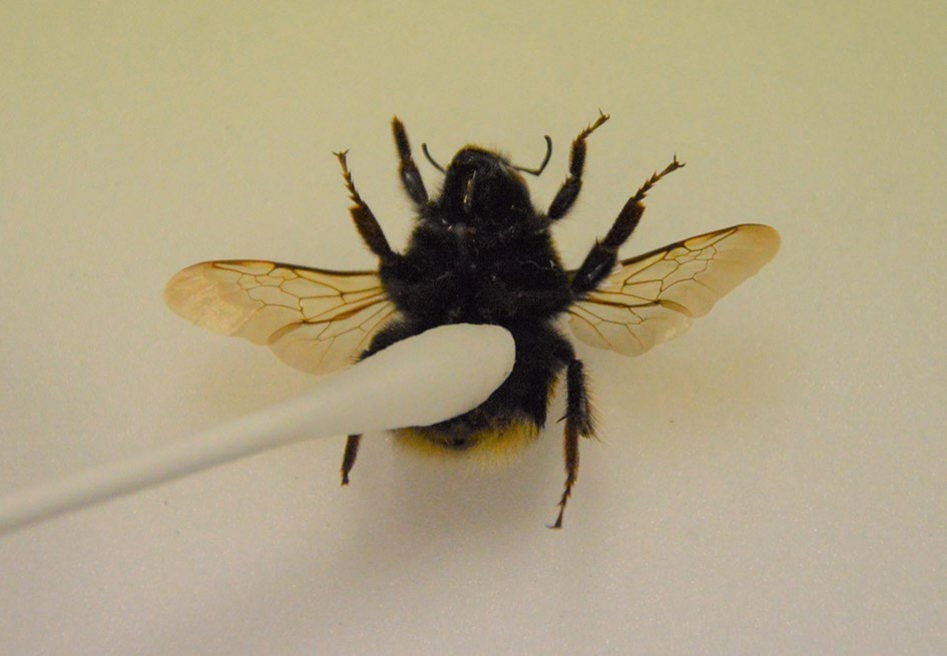
Application of the proposed extraction method on the *Bombus terrestris* sternites.

The procedures were schematized in Fig. 4. A sterile microbiological swab, previously soaked in the digestion buffer for 5 min, was gently rubbed twenty times (in a total of 2 min) over the sternites of each investigated bee (Fig. 5). Due to the removal of locality and identification tags resulted very complicated, it was chosen to work on the sternites because these areas usually have few diagnostic characters related to hair. This procedure makes the proposed method even more specifically to avoid visually damaging the specimens.

Each swab was soaked in a 2 ml microtube, filled with 1 ml digestion buffer, and incubated for 18–22 h at 56 °C with gentle agitation. After the treatment, a sterile microbiological swab soaked with 100% EtOH was gently rubbed several times over the sternites to stop further digestion. Finally, the individuals were air-dried and replaced back in the collections.

Before DNA extraction, the swabs were removed from each 2 ml microtube, and DNA purification was performed using a phenol:chloroform extraction (Ultrapure™ Phenol:Chloroform:Isoamyl Alcohol, ThermoFisher Scientific, Waltham, MA, USA), following Gilbert et al*.*^13^*.* Briefly, 20 µg glycogen, 0.6 volumes 100% isopropanol and 0.1 volumes 3 M Sodium acetate (pH 5.2) were added, and the microtubes were immediately vortexed softly and centrifuged at room temperature at maximum speed (1400 g) for 30 min to collect DNA as a pellet. The supernatant was then removed, and the pellet was washed twice in 1.5 ml 85% ethanol, air-dried at 65 °C, and resuspended in 100 µl RNase-DNase-free water. The obtained DNAs were quantified using the spectrophotometer Infinite 200 PRO NanoQuant™ (TECAN Life Technologies, Männedorf, Switzerland) and placed at − 20° until the analysis. For all of these processes, double-distilled RNase-DNase-free water was used as the negative control.

The extracted DNAs were analysed by PCR to amplify the *mt*DNA region. Primers amplified a 710-bp fragment within the highly conserved region coding for the *Cytochrome C oxidase subunit I* (*COI*) gene: *LCO1490* (5′-GGTCAACAAATCATAAAGATATTGG-3′) and *HC02198* (5′-TAAACTTCAGGGTGACCAAAAAATCA-3)^22^. The PCR was performed in 25 µl of volume using HotStarTaq Polymerase (Qiagen, Hilden, Germany) following manufacturers’ instructions using 5 µl of DNA, forward and reverse probes (500 nM). The PCR assay was performed on Applied Biosystems^®^ 2720 Thermal Cycler (ThermoFisher Scientific) and samples were amplified, after an initial activation at 95 °C for 15 min, through 35 cycles (1 min at 95 °C, 1 min at 40 °C, and 1.5 min at 72 °C), followed by a final extension at 72 °C for 7 min. All amplicons were visualized on a 1.5% agarose gel. The obtained amplicons were purified using ExoSAP-IT *Express* (ThermoFisher Scientific) and they were sequenced throughout the standard Sanger methodology (BMR Genomics, Padua, Italy). The obtained sequences were analysed using BioEdit^23^ to create the consensus one aligning forward and reverse sequences and BLAST (using megablast algorithm)^24^ and compared to sequences deposited in the Barcode of Life Data Systems (BOLD Systems)^25^.

## Data Availability

DNA sequences were deposited in GenBank (OL961135, OL966966, OL966999, OL967010, OL979169-OL979174, OL979211-OL979218, OL982531-OL982538, OL984023-OL984030; OL986022-OL986029).
